# High CIP2A immunoreactivity is an independent prognostic indicator in early-stage tongue cancer

**DOI:** 10.1038/bjc.2011.167

**Published:** 2011-05-24

**Authors:** C Böckelman, J Hagström, L K Mäkinen, H Keski-Säntti, V Häyry, J Lundin, T Atula, A Ristimäki, C Haglund

**Affiliations:** 1 Genome-Scale Biology Research Program, Biomedicum Helsinki, University of Helsinki, Haartmaninkatu 8, P.O. Box 63, 00014 Helsinki, Finland; 2 Department of Pathology, Haartman Institute, University of Helsinki, P.O. Box 21, Haartmaninkatu 3, 00014 Helsinki, Finland; 3 Department of Surgery, Helsinki University Central Hospital, P.O. Box 340, 00029 HUS, Helsinki, Finland; 4 Department of Oral Pathology, Institute of Dentistry, University of Helsinki, P.O. Box 41, Mannerheimintie 172, 00014 Helsinki, Finland; 5 Department of Pathology, HUSLAB, Helsinki University Central Hospital, P.O. Box 400, Haartmaninkatu 3, 00029 HUS, Helsinki, Finland; 6 Department of Otorhinolaryngology and Head and Neck Surgery, Helsinki University Central Hospital, P.O. Box 220, Haartmaninkatu 4E, 00029 HUS, Helsinki, Finland; 7 Institute for Molecular Medicine Finland, P.O. Box 20, 00014 Helsinki, Finland

**Keywords:** CIP2A, prognosis, survival, tongue cancer

## Abstract

**Background:**

No reliable prognostic markers exist for squamous cell carcinoma of the tongue, and its prognosis can even in early stages be unpredictable and survival poor despite treatment. A potential marker is oncoprotein cancerous inhibitor of PP2A (CIP2A), which acts as a prognostic marker in gastric and non-small cell lung cancers.

**Methods:**

We collected specimens of 73 stage T1N0M0 and T2N0M0 oral squamous cell carcinomas of the tongue, as well as samples from normal oral mucosa, dysplastic lesions, and invasive carcinomas (*n*=39). All samples were stained for CIP2A by immunohistochemistry. Survival curves were constructed according to the Kaplan–Meier method. The Cox proportional hazard model served for univariate and multivariate survival analysis.

**Results:**

High CIP2A immunoreactivity predicted poor survival in tongue cancer patients (*P*=0.027, logrank test). In multivariate survival analysis, CIP2A was an independent prognostic factor (HR 2.02, 95% confidence interval 1.07–3.82, *P*=0.030). Cytoplasmic CIP2A expression was higher in severe dysplasia than in mild dysplasia.

**Conclusion:**

Our results suggest that high CIP2A expression characterises aggressive disease. Acting as a prognostic marker it might be of help when choosing patients for adjuvant treatment in tongue cancer patients.

Oral squamous cell carcinoma, the most common cancer of the head and neck region, has, globally, a greatly varying incidence depending on geographical area and prevalent risk factors (Barnes *et al*, 2005; Curado *et al*, 2008). The increasing incidence of oral cancer is largely explained by a rise in tobacco or alcohol consumption, or both (Blot *et al*, 1988). Tongue cancer can be associated with an unpredictable prognosis and poor survival despite treatment (Bello *et al*, 2010a, 2010b). Its most important prognostic factors are tumour size, nodal involvement, and depth of infiltration, but the results concerning histological grade vary (Woolgar, 2006). Better prognostic markers are still needed, as survival for patients with the same clinicopathological stage varies considerably.

Cancerous inhibitor of PP2A (CIP2A) is an oncoprotein expressed in several cancers, among them head and neck (Junttila *et al*, 2007). It has a prognostic role in gastric and non-small cell lung cancers (Khanna *et al*, 2009; Dong *et al*, 2011); in addition, it is abundantly expressed in oral dysplastic and malignant tissues (Katz *et al*, 2010).

In tongue cancer, what is of particular importance is to predict relative risk for an individual patient in order to separate out those at high risk for recurrence to receive adjuvant treatment. This is especially demanding, as even small tumours occasionally metastasise (Keski-Säntti *et al*, 2007). On this basis, we studied the prognostic role of CIP2A expression in a series of early-stage (T1N0M0 and T2N0M0) oral tongue squamous cell carcinomas.

## Patients and methods

### Patients

We collected retrospectively 73 consecutive patients, preoperatively staged as T1N0M0 and T2N0M0 tumours, treated with curative intent for oral squamous cell carcinoma of the tongue. Treatment was given between 1992 and 2002 at the Helsinki University Central Hospital according to the guidelines of the tumour board meeting. The patient material has been described in detail by Keski-Säntti *et al* (2006, 2007). In brief, 36 men and 37 women were included, median age was 59 years (range 23–95). Initially, 35 (48%) tumours were classified as T1, and 38 (52%) as T2. Following resection for cure of that primary tumour, 31 patients received no further treatment, whereas 42 underwent elective neck treatment (neck dissection, 9; neck dissection and radiotherapy, 32; radiotherapy only, 1). In elective neck dissection, primary lymph-node positivity (pN+) was noted in 14. During follow-up, 10 developed neck recurrence apparently representing late lymph-node metastases without primary recurrence. Median follow-up of patients at study end was 7.9 years (range 0.3–17.2). Five-year overall survival was 68.5% (95% confidence interval (CI) 57.9–79.1). Approval of the study came from the local Ethics Committee and National Supervisory Authority of Welfare and Health. Survival data and cause of death were obtained before this study from patient records, the Population Registry, and Statistics Finland. All samples were reviewed by an oral pathologist.

### Tissue samples

Tumour samples were fixed in formalin, embedded in paraffin, and stored in the archives of the Department of Pathology, Helsinki University Central Hospital. Representative areas of each tumour were chosen from H&E staining of the tumour samples. Six representative 1 mm cores from marked areas were obtained from each tumour with a tissue microarray instrument (Beecher Instruments, Silver Spring, MD, USA) as described (Kononen *et al*, 1998; Kallioniemi *et al*, 2001; Torhorst *et al*, 2001). In nine patients, all array cores were missing or included no tumour tissue. In seven of these patients, whole sections from the tumour specimens were stained. Two cases had no representative tissue available for analysis.

In addition, representative specimens were collected from oral normal mucosa, dysplastic lesions, and invasive carcinoma (*n*=37).

### Immunohistochemistry

For the detailed immunohistochemistry protocol, see Khanna *et al* (2009). For antigen retrieval, slides were treated in a PreTreatment module (Lab Vision Corp., Fremont, CA, USA) in Tris–HCl buffer (pH 8.5) for 20 min at 98°C. Staining of sections was performed in Autostainer 480 (Lab Vision Corp.) using the Dako REAL EnVision Detection System, Peroxidase/DAB+, Rabbit/Mouse (Dako, Glostrup, Denmark). A rabbit polyclonal CIP2A antibody served, at a dilution of 1 : 3000 for 1 h at room temperature, as the primary antibody (Soo Hoo *et al*, 2002). The staining protocol for Ki-67 has been described (Häyry *et al*, 2010).

### Scoring of immunoreactivity

Tumour specimens were scored independently from whole sections (hot-spot areas) and tissue microarrays by two researchers (CB and JH) blinded to clinical status and outcome data. Cytoplasmic CIP2A immunopositivity was scored as 0–3 based on the intensity of cancer-cell immunoreactivity, and the highest intensity of the six cores was used for further analysis. Negative immunoreactivity was scored as 0, and diffuse weak cytoplasmic positivity as 1. Moderately positive or focally strongly positive intensity was scored as 2, and homogeneously strong intensity as 3. In normal mucosa, dysplastic lesions, and invasive carcinoma specimens, cytoplasmic CIP2A immunoreactivity was scored as described, and nuclear CIP2A immunoreactivity was evaluated for trend. Specimens with discordant scores underwent re-evaluation with a multiheaded microscope, and the consensus score served for further analysis. For survival analysis, we were able to score 71 (97%) patient samples for CIP2A. For statistical analysis, the patients were divided into two groups: low CIP2A immunoreactivity (scores 0–2) and high immunoreactivity (score 3).

### Statistical analysis

Associations between CIP2A positivity and clinicopathologic variables were assessed by the *χ*^2^-test. Overall survival was calculated from date of diagnosis to death, while disease-specific survival was calculated from date of diagnosis to death from tongue cancer. Survival curves were constructed according to the Kaplan–Meier method and compared with the logrank test (SPSS version 17.0 for Mac; SPSS, Inc., Chicago, IL, USA). For univariate and multivariate survival analysis, the Cox proportional hazard model had the following categorical covariates entered in a backward stepwise manner: tumour size (pT-classification), grade, invasion depth of the tumour, Ki-67 expression, and CIP2A expression.

## Results

### Immunohistochemistry and prognosis

**Figure 1:**
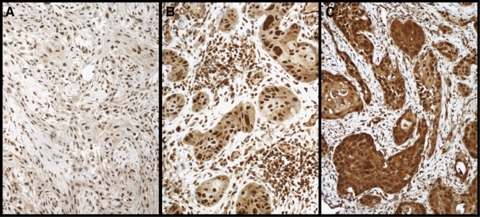
Cytoplasmic CIP2A expression in tongue cancer specimens was scored as (**A**) negative, (**B**) moderately positive, and (**C**) strongly positive. Original magnification was × 200.

**Figure 2:**
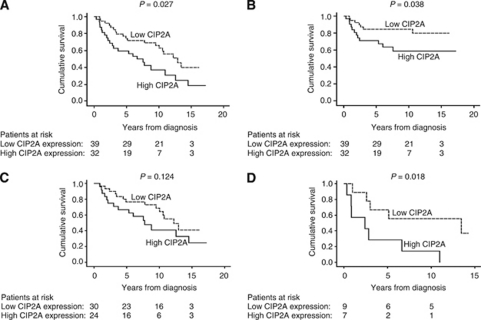
CIP2A expression and survival in tongue cancer patients. (**A**) Overall survival analysis according to the Kaplan–Meier method for cytoplasmic CIP2A immunoreactivity (logrank test, *P*=0.027). (**B**) Disease-specific overall survival analysis for cytoplasmic CIP2A immunoreactivity (logrank test, *P*=0.038). (**C**) Overall survival analysis stratified for patients with pT1 tumours (logrank test, *P*=0.124), and (**D**) pT2 tumours (logrank test, *P*=0.018).

Cytoplasmic CIP2A immunoreactivity, tested in 73 cases, was negative in 2 (2.8%), weakly positive in 9 (12.7%), moderately positive in 28 (39.4%), and strongly positive in 32 (45.1%) cases. In the final analysis, negative to moderately positive CIP2A immunoreactivity (scores 0–2) was regarded as representative of low expression, whereas strongly positive CIP2A (score 3) represented high expression. Representative images of immunostaining are shown in Figure 1. High cytoplasmic CIP2A immunoreactivity was a marker of reduced overall survival with a 5-year survival of 59.4% (95% CI 42.4–76.4) for the patients with strongly positive CIP2A, compared with 74.4% (95% CI 60.7–88.1) for those with low CIP2A expression (logrank test, *P*=0.027; Figure 2A). For disease-specific survival, 5-year survival for patients with strongly positive CIP2A was 71.0% (95% CI 55.0–87.0) compared with 84.6% (95% CI 73.3–96.0) for patients with low CIP2A immunoreactivity (logrank test, *P*=0.038; Figure 2B).

### Association of CIP2A with clinicopathological variables

An association appeared between high CIP2A expression and high histological tumour grade (*P*=0.009), and invasion depth >4 mm (*χ*^2^-test, *P*=0.041; Table 1). High CIP2A expression also associated with high proliferation index of the tumour (Ki-67, *P*=0.008), but no association appeared between cytoplasmic CIP2A immunoreactivity and age, gender, tumour size (pT-classification), or lymph-node positivity (pN-classification). We compared the tumours tendency to develop primary or late lymph-node metastases (lymph-node positivity in pathological examination or neck recurrence without a local recurrence) with CIP2A expression, and found no association between these (*P*=0.064).

### Univariate and multivariate survival analysis

Univariate survival analysis was performed for clinically important subgroups of tongue cancer patients. Patients with pT1 tumours showed no difference in overall survival when the analysis was stratified according to CIP2A expression (logrank test, *P*=0.124; Table 2; Figure 2C). Among patients with pT2 tumours, however, 5-year survival of CIP2A strongly positive patients was 28.6% (95% CI 0–62.0) compared with 66.7% (95% CI 35.9–97.5) for those with low CIP2A expression (logrank test, *P*=0.018; Figure 2D). In patients under 60 at diagnosis, CIP2A was a marker for poor prognosis with a 5-year survival of 58.8% (95% CI 35.4–82.2) for CIP2A strongly positive patients compared with 84.2% (95% CI 67.8–100.1) for those with low CIP2A immunoreactivity (logrank test, *P*=0.042; Table 2).

In multivariate survival analysis, age over 60 years (hazard ratio (HR) 2.18, 95% CI 1.11–4.28, *P*=0.023), tumour size 21–40 mm (HR 2.48, 95% CI 1.19–5.17, *P*=0.016), and high CIP2A expression (HR 2.02, 95% CI 1.07–3.82, *P*=0.030; Table 3) remained as independent prognostic factors.

### CIP2A expression in oral squamous epithelial lesions

**Figure 3:**
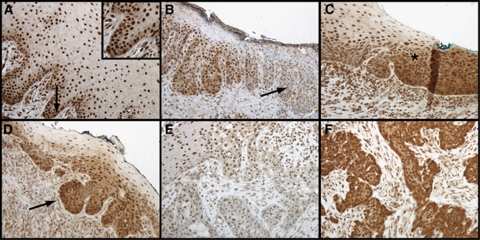
Characterisation of CIP2A expression during development of oral squamous epithelial dysplasia. (**A**) Normal oral mucosa with basal cytoplasmic CIP2A positivity (arrow) serving as a control specimen. (**B**) Severe dysplasia where nuclear positivity disappears (arrow) in oral buccal mucosa. (**C**) Transition area (asterisk) to carcinoma *in situ* from the anterior palatinal mucosa. (**D**) Epithelial budding (arrow) in mucosal tissue transforming into carcinoma. (**E**) Mild and (**F**) strong CIP2A immunoexpression in invasive oral squamous cell carcinoma.

We also studied CIP2A expression in a small series (*n*=37) of oral squamous epithelial lesions. In normal oral mucosa, the epithelium was negative or weakly positive for cytoplasmic CIP2A in the basal cell area, whereas nuclei tended to stain homogenously positive (Table 4; Figure 3A). As a trend, cytoplasmic CIP2A expression was higher in severe epithelial dysplasia than in mild dysplasia, compared with nuclear CIP2A, which was low in severe dysplasia but was expressed to a greater extent in lesions with mild dysplasia (Figure 3B–D). In invasive carcinomas, cytoplasmic CIP2A protein expression was either low (Figure 3E) or high (Figure 3F).

## Discussion

No reliable prognostic biomarkers for tongue cancer are in clinical use. We found here in oral squamous cell carcinoma of the tongue, high CIP2A expression to predict poor survival, with these patients’ 5-year overall survival being 59.4% compared with 74.4% for patients with low CIP2A expression. For disease-specific survival – with a 5-year survival of 71.0% *vs* 84.6% for those with low CIP2A expression – the result was similar. These results are in line with previous ones regarding gastric cancer (Khanna *et al*, 2009), and with findings of Dong *et al* (2011) on non-small cell lung cancer.

High CIP2A expression was able to predict poor prognosis within clinically important subgroups of tongue cancer patients, such as in pT2 tumours and in young patients. To date, in tongue cancer, stage is the most common prognostic factor used, but prognosis varies considerably even among similar tumours; and hence, we need better prognostic markers to avoid unnecessary adjuvant treatment for those with a favourable prognosis (Bello *et al*, 2010a, 2010b). Our results suggest a role for CIP2A as a prognostic marker in these pT2 and young patient groups, in which occult metastases are common despite small tumour size at first presentation (Keski-Säntti *et al*, 2007).

Junttila *et al* (2007) have shown that CIP2A promotes early cellular transformation and malignant growth in head and neck squamous cell carcinoma cells. We found an association between high CIP2A expression and high grade, tumour invasion depth >4 mm, and high proliferation index. These results indicate that CIP2A is a marker of aggressive disease, in line with our study on gastric cancer showing CIP2A expression to associate with high proliferation index and aneuploidy (Khanna *et al*, 2009). In breast cancer, Come *et al* (2009) showed that high CIP2A expression associates with high proliferation index, p53 mutation, and high Scarff–Bloom–Richardson grade. In lung cancer, Dong *et al* (2011) demonstrated CIP2A to be an independent prognostic factor. In our present study we show that CIP2A is an independent prognostic factor in tongue cancer.

We found no association between CIP2A and cytoplasmic or nuclear c-Myc immunoexpression in tongue cancer (data not shown), when we compared CIP2 with cytoplasmic and nuclear c-Myc immunoreactivity previously described (Häyry *et al*, 2010), in contrast to our results in gastric cancer (Khanna *et al*, 2009). In lung cancer, a correlation is detectable between CIP2A and c-Myc mRNA expression levels (Dong *et al*, 2011). It is possible that CIP2A and c-Myc have different expression levels in tongue cancer than in other cancers studied before.

In hepatocellular carcinoma, CIP2A is found to mediate PP2A-dependent Akt inactivation (Chen *et al*, 2010). In preneoplastic lesions, p-Akt is frequently activated, and it serves as a prognostic marker for poor disease-free survival in tongue cancer (Massarelli *et al*, 2005). The possible route by which CIP2A affects the aggressiveness and poor outcome in tongue cancer may be through the Akt signalling pathway. Another possible signalling pathway of CIP2A influence may be via the death-associated protein kinase (DAPk) protein. Recently, Guenebeaud *et al* (2010) discovered that CIP2A inhibits DAPk-mediated apoptosis and hence, may increase malignant growth (Gozuacik and Kimchi, 2006). In head and neck cancer, hypermethylation at the *DAP-kinase* gene promoter correlated with advanced disease and lymph-node metastases (Sanchez-Cespedes *et al*, 2000).

In comparison with lung and gastric cancers, in tongue cancer CIP2A expression is more intense. Only two specimens were scored as completely negative, with 85% of the specimens scored as moderately or strongly positive (scores 2 and 3). The difference in level of expression might be due to tongue cancer's being an epidermoid cancer, whereas previously studied cancers were mainly adenocarcinomas (Junttila *et al*, 2007; Khanna *et al*, 2009; Dong *et al*, 2011). Moreover, in esophageal squamous cell carcinoma, CIP2A was overexpressed in 90% of the specimens studied (Qu *et al*, 2010).

Cancerous inhibitor of PP2A is overexpressed in human oral dysplasia and carcinoma tissues compared with expression in normal oral mucosa (Katz *et al*, 2010). Interestingly, in comparing severe dysplasia with mild dysplasia, our impression was that a gradual decrease in the amount of nuclear CIP2A staining and simultaneously an increasing cytoplasmic staining is seen. The limited number of specimens, however, did not allow statistical analysis. The role of nuclear and cytoplasmic CIP2A remains thus far unresolved. In cancer specimens, however, we noted that cytoplasmic CIP2A was expressed both at low and at high levels. Junttila *et al* (2007) have suggested that CIP2A expression is already induced in premalignant head and neck squamous cell carcinoma tissue in response to a combination of oncogenic Ras signalling with inhibition of the TGF-*β* tumour suppressor pathway.

In conclusion, in tongue cancer, high cytoplasmic CIP2A expression characterises aggressive disease and is an independent prognostic marker indicating need for adjuvant treatment after surgery. Our results encourage further validation and quantification of the scoring methods, as a step towards developing CIP2A into a clinically useful biomarker in routine pathology.

## Figures and Tables

**Table 1:** Association of CIP2A with clinicopathologic variables in 71 tongue squamous cell carcinoma patients

		**Low CIP2A expression**		**High CIP2A expression**		
**Clinicopathological variable**	***n***	***n***	**%**	***n***	**%**	***P*-value^a^**
*Age, years*						
<60	36	19	52.8	17	47.2	0.712
⩾60	35	20	57.1	15	42.9	
						
*Gender*						
Male	37	18	48.6	19	51.4	0.267
Female	34	21	61.8	13	38.2	
						
*Grade*						
I	23	18	78.3	5	21.7	0.009
II	34	17	50.0	17	50.0	
III	14	4	28.6	10	71.4	
						
*Tumour size, mm*						
pT1 (⩽20)	54	30	55.6	24	44.4	0.961
pT2 (21–40)	16	9	56.3	7	43.8	
						
*Node positivity*						
pN0	26	16	61.5	10	38.5	0.119
pN+	14	5	35.7	9	64.3	
						
*Invasion depth, mm*						
⩽4	27	19	70.4	8	29.6	0.041
>4	44	20	45.5	24	54.5	
						
*Ki-67, %*						
0–29	13	8	61.5	5	38.5	0.008
30–49	23	18	78.3	5	21.7	
50–79	14	6	42.9	8	57.1	
⩾80	16	4	25.0	12	75.0	

Abbreviation: CIP2A=cancerous inhibitor of PP2A.

a *χ*^2^-test.

**Table 2:** Survival analysis according to CIP2A immunoreactivity in subgroups of tongue cancer

	**Low CIP2A expression**		**High CIP2A expression**		
**Characteristic**	**5-year survival**	**95% CI**	**5-year survival**	**95% CI**	***P*-value^a^**
*Age, years*					
<60	84.2	67.8–100.1	58.8	35.4–82.2	0.042
⩾60	65.0	44.1–85.9	60.0	35.2–84.8	0.347
					
*Tumour size, mm*					
pT1 (⩽20)	76.7	61.5–91.8	66.7	47.8–85.5	0.124
pT2 (21–40)	66.7	35.9–97.5	28.6	0–62.0	0.018

Abbreviations: CIP2A=cancerous inhibitor of PP2A; CI=confidence interval.

a Logrank test.

**Table 3:** Cox regression analysis for overall survival of tongue cancer patients

	**Univariate survival analysis**			**Multivariate survival analysis**		
**Variable**	**Hazard ratio**	**95% CI**	***P*-value**	**Hazard ratio**	**95% CI**	***P*-value**
*Age, years*						
<60	1.00					
⩾60	1.84	0.96–3.45	0.056	2.18	1.11–4.28	0.023
						
*Gender*						
Male	1.00					
Female	0.97	0.52–1.79	0.916			
						
*Grade*						
I	1.00					
II	1.95	0.93–4.11	0.079			
III	1.88	0.74–4.81	0.185			
						
*Tumour size, mm*						
pT1 (⩽20)	1.00					
pT2 (21–40)	1.10	0.97–3.76	0.062	2.48	1.19–5.17	0.016
						
*Invasion depth, mm*						
⩽4	1.00					
>4	2.20	1.10–4.40	0.025			
						
*Ki-67, %*						
0–29	1.00					
30–49	1.30	0.53–3.18	0.561			
50–79	1.44	0.52–4.00	0.483			
⩾80	1.23	0.46–3.33	0.682			
						
*CIP2A expression*						
Low	1.00					
High	1.99	1.07–3.70	0.030	2.02	1.07–3.82	0.030

Abbreviations: CIP2A=cancerous inhibitor of PP2A; CI=confidence interval.

**Table 4:** Distribution of scores in normal, dysplastic, and invasive carcinoma specimens (*n*=37)

	**CIP2A cytoplasmic score (*n*)**				
**Tumour type**	**0**	**1**	**2**	**3**	**Total**
Normal oral epithelium	1	1	1	0	3
Mild dysplasia	0	4	1	0	5
Moderate dysplasia	0	1	0	0	1
Severe dysplasia	0	2	3	1	6
*In situ* carcinoma	0	4	4	0	8
Microinvasive carcinoma	0	2	2	0	4
Squamous cell carcinoma	0	1	6	3	10

Abbreviation: CIP2A=cancerous inhibitor of PP2A.
